# Origin and consequences of brain Toll-like receptor 4 pathway stimulation in an experimental model of depression

**DOI:** 10.1186/1742-2094-8-151

**Published:** 2011-11-03

**Authors:** Iciar Gárate, Borja García-Bueno, José LM Madrigal, Lidia Bravo, Esther Berrocoso, Javier R Caso, Juan A Micó, Juan C Leza

**Affiliations:** 1 Department of Pharmacology, Faculty of Medicine, Universidad Complutense, Madrid 28040, Spain; 2 Department of Psychiatry, Faculty of Medicine, Universidad Complutense, Madrid 28040, Spain; 3 Department of Neurosciences, Faculty of Medicine, Universidad de Cádiz, Cádiz 11003, Spain; 4 Centro de Investigación Biomédica en Red de Salud Mental (CIBERSAM), Spain; 5 Instituto de Investigación Sanitaria Hospital 12 de Octubre, Madrid 28026, Spain

**Keywords:** neuroinflammation, chronic mild stress, depression, innate immunity, TLR-4, LPS

## Abstract

**Background:**

There is a pressing need to identify novel pathophysiological pathways relevant to depression that can help to reveal targets for the development of new medications. Toll-like receptor 4 (TLR-4) has a regulatory role in the brain's response to stress. Psychological stress may compromise the intestinal barrier, and increased gastrointestinal permeability with translocation of lipopolysaccharide (LPS) from Gram-negative bacteria may play a role in the pathophysiology of major depression.

**Methods:**

Adult male Sprague-Dawley rats were subjected to chronic mild stress (CMS) or CMS+intestinal antibiotic decontamination (CMS+ATB) protocols. Levels of components of the TLR-4 signaling pathway, of LPS and of different inflammatory, oxidative/nitrosative and anti-inflammatory mediators were measured by RT-PCR, western blot and/or ELISA in brain prefrontal cortex. Behavioral despair was studied using Porsolt's test.

**Results:**

CMS increased levels of TLR-4 and its co-receptor MD-2 in brain as well as LPS and LPS-binding protein in plasma. In addition, CMS also increased interleukin (IL)-1β, COX-2, PGE_2 _and lipid peroxidation levels and reduced levels of the anti-inflammatory prostaglandin 15d-PGJ_2 _in brain tissue. Intestinal decontamination reduced brain levels of the pro-inflammatory parameters and increased 15d-PGJ_2_, however this did not affect depressive-like behavior induced by CMS.

**Conclusions:**

Our results suggest that LPS from bacterial translocation is responsible, at least in part, for the TLR-4 activation found in brain after CMS, which leads to release of inflammatory mediators in the CNS. The use of Gram-negative antibiotics offers a potential therapeutic approach for the adjuvant treatment of depression.

## Background

The complete remission of symptoms, while not the cure, is the goal of treatment of any disease, but in neuropsychiatric disorders (such as depression) patients frequently fail to maintain a long-term symptom-free status [1,2]. When depression does not respond adequately to treatment with an antidepressant, clinicians should be able to choose different strategies including adding another compound to the pharmacological treatment or other non-pharmacological strategies. However, despite advances in our understanding of depression, resistance is still a significant challenge for clinicians and their patients, with non-response in at least one-third of cases [3]. Exposure to external stressors is widely acknowledged as a predisposing and precipitating factor of depression, and an increasing body of evidence presented in recent years has shown that exposure to certain psychological experiences, including stress-induced diseases, is associated with variations in immune parameters. In some cases both depression and chronic stressors have been associated with decreased adaptative/adquired immunity and inflammation but it has been only recently demonstrated that after stress exposure or during certain episodes of depression an innate inflammatory/immune response is strongly activated [4-7]. A matter of special relevance is that, although the brain has long been considered to be an "immune-privileged" organ, this immune status is far from absolute, especially when blood-brain barrier (BBB) structure or function may be affected, as is the case after stress exposure in animal models of depression or in humans with depression [8-12].

The brain monitors peripheral immune responses by several means acting in parallel [6]: some involve locally produced cytokines or pro-inflammatory cytokine transporters at the BBB and cells surrounding the perivascular space; in another humoral pathway, Toll-like receptors (TLRs) on macrophage-like cells residing in the CNS respond to circulating pathogen components by producing pro-inflammatory cytokines and other pro-inflammatory mediators.

Recently, several studies have focused on TLRs and their potential roles in neuropathology [13]. The discovery that not only immune cells, but also neurons, astrocytes and resident microglia express a large majority of the already discovered 10 TLRs has challenged the way neuroscience explains the role of the immune system in the brain and, as a result, the view of the brain as an immune privileged organ has been re-evaluated.

TLRs are pattern recognition receptors. Their expression is not static, being rapidly modulated in response to pathogens, a variety of cytokines, and environmental stresses [14]. One of these, TLR-4, has been reported to have a regulatory role in the adrenal response to stressful inflammatory stimuli as well as in the brain's response to stress [15,16]. TLR-4 responds predominantly to lipopolysaccharide (LPS) from Gram-negative bacteria. To achieve specificity of signaling, TLRs recruit some co-receptors such as, in the case of TLR-4, the myeloid differentiation factor MD-2. After various steps in the transduction pathway (i.e. specific kinases), the signal leads to activation of the prototypic inflammatory nuclear transcription factor NF-κB and others such as AP-1 [14]. Activation of NF-κB culminates in production of NF-κB-dependent pro-inflammatory mediators, such as the products of the inducible isoforms of the enzymes nitric oxide synthase (iNOS) and cyclooxygenase (COX-2). This cellular pathway has been described in brain cells (neurons and glia) where inflammatory and oxidative-nitrosative damage takes place after stress exposure and in humans with depression [5,17-19].

Two major mechanisms have been proposed to activate TLR-4 after immune/inflammatory stimuli (stress exposure included): the first is related to endogenous molecules or DAMPs (damage-associated molecular patterns) released from disrupted cells and extracellular matrix degradation products that may contribute to immune activation and inflammation after tissue injury [20]. The second comes from models of stress that show increased intestinal permeability and resultant bacterial translocation to the systemic circulation [21,22]. These circulating Gram-negative enterobacteria are a major source of LPS, the main activator of TLR-4 expression in the CNS, inducing a neuroinflammatory response. This proposed mechanism, known as "*leaky gut*", also takes place in depressed patients and has been related to the inflammatory pathophysiology of the disease [23].

Thus, the aims of the present study were to evaluate (1) activation of the TLR-4 pathway in brain after chronic stress exposure, (2) the possible role of LPS, resulting from intestinal bacterial traslocation after stress, in this activation, and (3) the potential role of new pharmacological approaches to control stress-induced neuroinflammation. To accomplish these aims, we used a chronic mild stress model in rats widely accepted as an experimental model of depression.

## Methods

### Animals

Male Sprague-Dawley rats, initially weighing 200-220 g, were used. All animals were housed under standard conditions of temperature and humidity in a 12-hour-light/dark cycle (lights on at 08:00 h), with free access to food and water, and were maintained under constant conditions for 15 days prior to induction of stress. All experimental protocols adhered to the guidelines of the Animal Welfare Committee of the University of Cadiz following European legislation (2003/65/EC).

### Experimental groups

Four groups (n = 8-10 in each group) were used: (1) a control group (Control); (2) a chronic mild stress group (CMS); (3) a control group treated with antibiotics (Control+ATB) and (4) a chronic mild stress group treated with antibiotics (CMS+ATB). The antibiotic-treated groups were designed to test the possibility of Gram-negative LPS induction of TLR-4 caused by intestinal bacterial translocation after stress.

### Intestinal antibiotic decontamination

We followed a previously described protocol for rats [24]. Briefly, animals were given drinking water *ad libitum *containing streptomycin sulphate (2 mg/ml) and penicillin G (1,500 U/ml), from the first day of stress (at 08:00 h) until the moment of sacrifice, to reduce indigenous gastrointestinal microflora.

### Chronic mild stress and tissue samples

The CMS protocol used was a modification of the one proposed by Willner [25]. The protocol consists of a series of different stressors that were changed daily for a period of 21 days. The stressors included: (a) food deprivation, (b) water deprivation, (c) cage tilting, (d) soiled cage, (e) grouped housing after a period of water deprivation (f), stroboscopic illumination (150 flashes/min) and (g) intermittent illumination every 2 hours.

To avoid variations in corticosterone levels caused by circadian rhythms, all animals were sacrificed at the same time of day (15:00 h) and, specifically, CMS-exposed animals were killed immediately after the 21 days of stress, using chloral hydrate (400 mg/kg i.p.). Blood for plasma determinations was collected by cardiac puncture and anti-coagulated in the presence of tri-sodium citrate (3.15% w:v, 1 vol citrate per 9 vol blood). After decapitation, brains were removed from the skull and both cortical areas were excised from the brain and frozen at -80°C until assayed. Rat brain prefrontal cortex was chosen because of its high levels of pro-inflammatory (NF-κB, COX-2) mediators, its susceptibility to the neuroinflammatory process elicited by stress [5] and, finally, because this brain area is an important neural substrate for regulation of the hypothalamic/pituitary/adrenal (HPA) axis response to stress [26]. TLR-4 expression has been found after different immune/inflammatory challenges in murine primary cortical neurons, astrocytes, microglia and endothelial cells [27-30].

### Plasma corticosterone levels

Plasma was obtained from blood samples by centrifuging samples at 1500 g for 10 min immediately after stress. All plasma samples were stored at -40°C until assayed by means of a commercially available RIA (Coat-a-Count^®^, Siemens). The values obtained in basal conditions (182.9 ± 20.20 ng/mL) were in accordance with the values obtained in previous studies for adult rats at the time of blood extraction (15:00 h) [31].

### Behavioral studies

In order to verify depressive-like behavior, one set of animals (including control, CMS, control+ATB and CMS+ATB) was tested after 21 days of CMS exposure by the modified forced swimming test (mFST) based in the method described by Porsolt [32]. The mFST is by itself an important stressor; thus, we decided to use a different set of animals for behavioral studies after CMS.

Briefly, the rats were placed individually into plexiglas cylinders (height 40 cm, diameter 18 cm) filled with water (25 ± 1°C). Two different sessions were performed with a 15 min pre-test followed by a test of 5 min performed 24 hours later. The two sessions were assessed using a camera connected to a video tracking system. The time of climbing was measured when the rats made upward-directed movements of the forepaws along the side of the swim chamber. The time of swimming was measured when the rats showed active swimming movement throughout the swim chamber that also included crossing into another quadrant. Immobility was considered when the rats did not show additional activity other than movements necessary to keep their heads above water. Depressive-like behavior (behavioral despair) was defined as an increase in time of immobility. Some other physiological measures were taken: weight loss during the entire 21-day protocol and number of faecal boli during the test session.

### Plasma LPS (lipopolysaccharide) and LBP (lipopolysaccharide binding protein) levels

Plasma LPS and LBP levels were determined using commercially available kits following the manufacturer's instructions (Hycult Biotech, The Netherlands). Plasma LPS was measured using a chromogenic endpoint assay. The principle of the test is based on the fact that bacteria cause intravascular coagulation in the American horseshoe crab, *Limulus polyphemus*. Endotoxin causes an opacity and gelation in *Limulus *amebocyte lysate (LAL), which is based on an enzymatic reaction that cause a yellow color. LPS was measured at 450 nm in a spectrophotometer (Molecular Devices^®^). Results are expressed as endotoxin units (EU) per mL (EU/mL).

LPS binding protein (LBP) is a type 1 acute phase protein that is constitutively produced by the liver and rapidly up-regulated during the acute phase response. LBP plays a central role in the response to LPS by catalysing its monomerization and its transfer to receptors and lipoproteins. LBP was measured at 450 nm in a spectrophotometer (Molecular Devices^®^). The results are expressed as ng/mL of plasma.

### Western blot analysis

To determine expression levels of TLR-4, the TLR-4 co-receptor MD-2 (myeloid differentiation factor 2) and the inflammatory transcription factor NFκB subunit p65, brain prefrontal cortex was homogenized by sonication in 400 μl of PBS (pH = 7) mixed with a protease inhibitor cocktail (Complete, Roche^®^) followed by centrifugation at 12.000 g for 10 minutes at 4°C. After adjusting protein levels in the resultant supernatants, homogenates were mixed with Laemmli sample buffer (Bio Rad, Hercules, CA, USA) (SDS 10%, distilled H_2_O, glycerol 50%, Tris HCl 1 M pH 6,8, dithiotreitol and blue bromophenol). Then, 10 μl (1 mg/ml) were loaded and the proteins size-separated by 10% SDS-polyacrylamide gel electrophoresis (90 V). In the case of the NF-kB subunit p65, analyses were carried out on nuclear extracts (see next point).

Afterward the membranes were blocked in 30 ml Tris-buffered saline containing 0.1% Tween 20 and 5% skim milk/BSA; then the membranes were incubated with specific primary antibodies against p65, MD-2 and TLR-4 (Santa Cruz Biotechnology, 1:1000) and, after washing with a TBS-Tween solution, the membranes were incubated with the respective horseradish peroxidase-conjugated secondary antibodies for 90 min at room temperature and revealed by ECL™-kit following manufacturer's instructions (Amersham Ibérica, Spain). Autoradiographs were quantified by densitometry using ImageJ^® ^software and expressed as optical density (O.D.). Several exposition times were analyzed to ensure linearity of the band intensities, and the housekeeping proteins b-actin and sp-1 were used as loading controls for cytosolic and nuclear protein fractions, respectively (blots shown in the respective figures). Antibodies were from Santa Cruz, CA, USA, except for b-actin (from Sigma Spain).

### Preparation of cytosolic and nuclear extracts

In order to quantify the transcription factor NF-κB components, we used cytosolic or nuclear extracts. Activation of NF-κB occurs by enzymatic degradation of the bound inhibitory protein, predominantly IκBα, allowing movement of the p50/65 subunits from the cytoplasm to the nucleus where they bind to consensus κB sequences in DNA.

Tissues (brain frontal cortex) were homogenized in 300 μL of buffer [10 mmol/L *N*-2-hydroxyethylpiperazine-N-2-ethanesulfonic acid (pH 7.9); 1 mmol/L EDTA, 1 mmol/L EGTA, 10 mmol/L KCl, 1 mmol/L dithiothreitol, 0.5 mmol/L phenylmethylsulfonyl fluoride, 0.1 mg/ml aprotinin, 1 mg/mL leupeptin, 1 mg/mL Na-*p*-tosyll-lysine-chloromethyl ketone, 5 mmol/L NaF, 1 mmol/L NaVO_4_, 0.5 mol/L sucrose, and 10 mmol/L Na_2_MoO_4_]. After 15 minutes, Nonidet P-40 (Roche^®^, Mannheim, Germany) was added to reach a 0.5% concentration. The tubes were gently vortexed for 15 seconds, and nuclei were collected by centrifugation at 8000 *g *for 5 min. Supernatants were considered to be the cytosolic fraction. The pellets were resuspended in 100 ml buffer supplemented with 20% glycerol and 0.4 mol/liter KCl and gently shaken for 30 min at 4°C. Nuclear protein extracts were obtained by centrifugation at 13,000 *g *for 5 min, and aliquots of the supernatant were stored at -80°C. All steps of the fractionation were carried out at 4°C. As an analysis of purity, extracts were assayed against IκBα, sp-1 or b-actin (in cytosol: 83 ± 4; 19 ± 5; 98 ± 1 [% of total OD signal] respectively; in nuclei: 16 ± 9; 81 ± 7; 99 ± 1 [% of total OD signal] respectively).

### Nuclear factor *kappa *B (NF-κB) activity

The activity of nuclear factor κB was measured in nuclear extracts (obtained as described above) through a commercially available NF-κB (p65) Transcription Factor Assay (Cayman Chemicals, MI, USA) following the manufacturer's instructions. Briefly, a specific double-stranded DNA (dsDNA) sequence containing the NF-κB response element was immobilized to wells of a 96-well plate and nuclear extract was added. NF-κB (p65) was detected by addition of a specific primary antibody directed against it and a secondary antibody conjugated to HRP was added to provide a sensitive colorimetric readout at 450 nm. The plate was read in a spectrophotometer (BioTek^®^, Synergy 2). The optical density (O.D.) was normalized using the amount of protein present in the nuclear fraction - (O.D.)/mg of protein - and the results are presented as percentage of control.

### PCR analysis

Total cytoplasmic RNA was prepared from cells using Trizol^® ^reagent (Invitrogen, Carlsbad, CA, USA); aliquots were converted to cDNA using random hexamer primers. Quantitative changes in mRNA levels were estimated by real time PCR (Q-PCR) using the following cycling conditions: 35 cycles of denaturation at 95°C for 10 s, annealing at 58-61°C for 15 s depending on the specific set of primers, and extension at 72°C for 20 s. Reactions were carried out in the presence of SYBR green (1:10000 dilution of stock solution from Molecular Probes, Eugene, OR, USA), carried out in a 20-L reaction in a Rotor-Gene (Corbett Research, Mortlake, NSW, Australia).

The primers used were: for iNOS, forward: 5'-GGA CCA CCT CTA TCA GGA A-3', and reverse: 5'-CCT CAT GAT AAC GTT TCT GGC-3', for COX-2 forward: 5'-CTT CGG GAG CAC AAC AGA G-3', and reverse: 5'-GCG GAT GCC AGT GAT AGA G-3', for TLR4, forward: 5'-AGT TGG CTC TGC CAA GTC TCA GAT- 3', reverse: 5'-TGG CAC TCA TCA GGA TGA CAC CAT-3', for MD-2 forward: 5'-CAT AGA ATT GCC GAA GCG CAA GGA-3', reverse: 5'-ACA CAT CTG TGA TGG CCC TTA GGA-3', for NFκB subunit p65, forward: 5'-CAT GCG TTT CCG TTA CAA GTG CGA-3', reverse: 5'-TGG GTG CGT CTT AGT GGT ATC TGT-3', for IκBα forward: 5'-TGG CCT TCC TCA ACT TCC AGA ACA-3', reverse: 5'-TCA GGA TCA CAG CCA GCT TTC AGA-3', for tubulin, forward: 5'-CCC TCG CCA TGG TAA ATA CAT-3', reverse: 5'-ACT GGA TGG TAC GCT TGG TCT-3', for IL-1β, forward: 5'-ACC TGC TAG TGT GTG ATG TTC CCA-3', and reverse: 5'-AGG TGG AGA GCT TTC AGC TCA CAT-3'.

Relative mRNA concentrations were calculated from the take-off point of reactions using included software, and tubulin levels were used to normalize data.

### Lipid peroxidation

As a marker of reactive oxygen species attack to the lipidic components of a particular tissue, lipid peroxidation rates were measured in brain cortex homogenates using the thiobarbituric acid test for malonildialdehyde (MDA) following the method described by Das and Ratty with some modifications [33]. Briefly, cortical fragments were sonicated in 10 vol 50 mM phosphate buffer and deproteinised with 40% trichloroacetic acid and 5 M HCl, followed by the addition of 2% (w/v) thiobarbituric acid in 0.5 M NaOH. The reaction mixture was heated in a water bath at 90°C for 15 min and centrifuged at 12,000 g for 10 min. The pink chromogen was measured at 532 nm (BioTek^®^, Synergy 2). The results are expressed as nanomols per milligram (nmol/mg) of protein.

### Brain PGE_2 _levels

Prostaglandin E_2 _(PGE_2_) prefrontal cortex levels were determined using an enzyme immunoassay kit (Cayman Chemicals, MI, USA). PGE_2 _is known as one of the main inflammatory and oxido-nitrosative mediators in brain after multiple stimuli [34]. Samples were purified using polypropylene minicolumns C-18 (Waters Corp. MA, USA). Tissues were homogenized by sonication in ice-cold phosphate buffer (pH 7.4) containing EDTA (1 mM) and indomethacin (10 μM). Enzyme immunoassay isolation and prostaglandin quantification were carried out following manufacturer's instructions.

### Brain 15-deoxy-Δ^12,14^-PGJ_2 _levels

Prefrontal cortex levels of 15-deoxy-Δ^12,14^-prostaglandin J_2 _(15d-PGJ_2_) were determined using an enzyme immunoassay kit (DRG Diagnostics, Marburg, Germany). 15d-PGJ_2 _is the main component of the anti-inflammatory counterbalance mechanism in COX-containing cells [35]. Homogenization, purification of samples and quantification procedures were the same as for the PGE_2 _determination.

### Protein assay

Protein levels were measured using the Bradford method, based on the principle of protein-dye binding [36].

### Chemicals and statistical analyses

Unless otherwise stated, chemicals were from Sigma-Aldrich (Spain). Data in text and figures are expressed as mean ± SEM. For multiple comparisons, a one-way ANOVA followed by the Newman-Keuls *post hoc *test to compare all pairs of means between groups was made. When comparing only two experimental groups a two-tailed t-test was employed. Two-way analysis of variance (ANOVA) followed by a Bonferroni *post hoc *test was used for the statistical analysis of the forced swimming test. A *p *value < 0.05 was considered statistically significant.

## Results

### 1.- TLR-4 expression and signaling in brain cortex after CMS exposure

**Figure 1:**
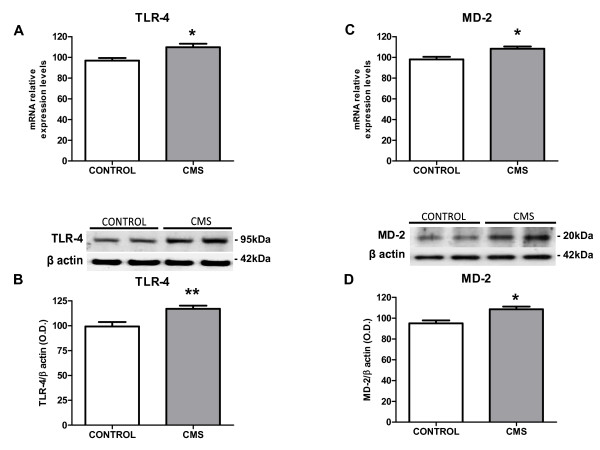
**TLR-4 pathway activation in brain cortex after stress exposure in rats**. mRNA expression levels for TLR-4 (A) and MD-2 (C) in brain in control and after CMS. Protein expression of TLR-4 (B) and MD-2 (D) in brain in control and after CMS. Data are mean ± SEM of 8-10 rats per group. * p < 0.05, ** p < 0.01 *vs*. Control group (two-tailed t-test).

To evaluate if the TLR-4 pathway is activated after stress exposure we studied the expression of TLR-4 and its co-receptor, myeloid differentiation factor-2 (MD-2). Stress exposure induced a significant increase in TLR-4 mRNA and protein levels in the brain cortex (Figure 1A&1B). Similarly, MD-2 was up-regulated after stress (Figure 1C&1D).

### 2.- Possible regulatory mechanisms of TLR-4 activation in brain cortex after CMS

**Figure 2:**
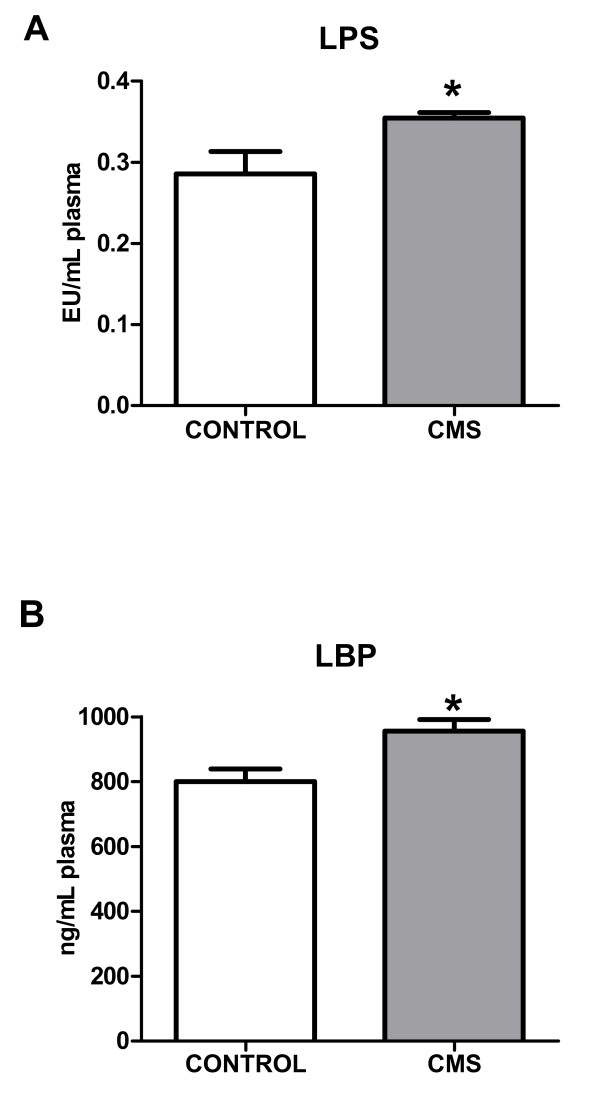
**LPS (A) and LBP (B) levels in plasma in control and after CMS**. Data are mean ± SEM of 8-10 rats per group. * p < 0.05 *vs*. Control group (two-tailed t-test).

Lipopolysaccharide (LPS) is a main ligand of TLR-4, whose activation switches on intracellular inflammatory pathways. In order to clarify the origin of the stress-induced activation of the TLR-4 pathway, we studied plasma levels of LPS and LPS binding protein (LBP). CMS exposure produced an increase in both LPS and LBP plasma levels (Figure 2A&2B).

### 3.- Inflammatory mediators in brain cortex after CMS exposure

**Figure 3:**
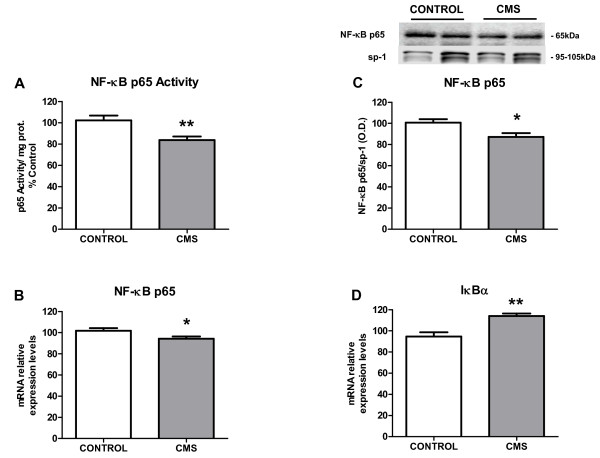
**NF-κB signaling in brain cortex after CMS exposure: p65 activity (A), p65 mRNA levels (B) and p65 protein expression (C) in nuclear fractions of brain cortex in control and CMS**. IκBα mRNA levels in cytoplasmic fractions of cortex in control and CMS (D). Data are mean ± SEM of 8-10 rats per group. * p < 0.05, ** p < 0.01 *vs*. Control group (two-tailed t-test).

TLR-4 activation is followed by stimulation of the pro-inflammatory transcription nuclear factor κB (NF-κB) [37], whose p65 subunit can be determined in cell nuclei to evaluate its activation (by cytoplasm-nuclear trafficking) after stress or other immune/inflammatory stimuli. Under the conditions used in this study, a decreased activity of NF-κB after CMS exposure was detected (Figure 3A). Similarly, a decrease in mRNA levels and protein expression of p65 subunit (Figure 3B&3C) was observed in nuclear fractions from brain cortex of stressed individuals as well. Stress also increased mRNA expression of the NF-κB inhibitory protein, IκBα in the cytoplasm (Figure 3D).

**Figure 4:**
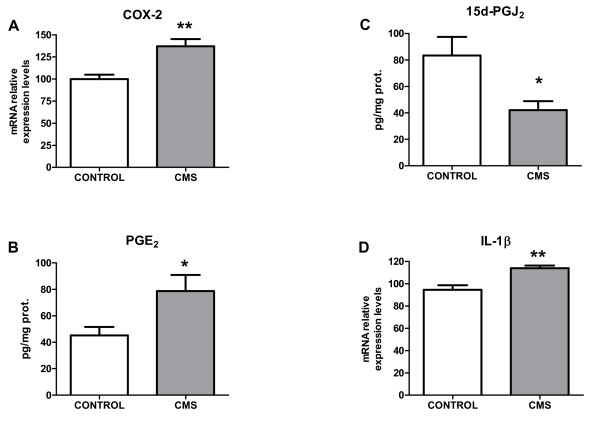
**Inflammatory parameters in brain cortex after CMS**. Protein expression of COX-2 in control and after CMS in the brain (A). Brain levels of the pro-inflammatory prostaglandin PGE_2 _(B), the anti-inflammatory one 15d-PGJ_2 _(C), and interleukin-1β (IL-1β) mRNA levels in control and after CMS in the brain (D). Data are mean ± SEM of 8-10 rats per group. * p < 0.05, ** p < 0.01 *vs*. Control group (two-tailed t-test).

The pro-inflammatory enzymatic source inducible cyclooxygenase (COX-2) was also assessed in control and after stress-exposure conditions. An increase in COX-2 mRNA and in levels of its main product in brain, PGE_2 _was observed after 21 days of chronic stress (Figures 4A&4B). Taking into account that inflammation is a regulated process, we decide to study the main component of the anti-inflammatory mechanism: levels of 15-deoxy-Δ^12,14^-prostaglandin J_2 _(15d-PGJ_2_), an anti-inflammatory product of COX-2, were decreased in prefrontal cortex after CMS exposure (Figure 4C).

Another well known inflammatory agent in brain that is activated after TLR-4 activation is the pro-inflammatory cytokine IL-1β [6]. In this particular stress model, an increase in IL-1β mRNA levels was also detected (Figure 4D).

### 4.- Oxidative/nitrosative damage in brain cortex after CMS exposure

**Figure 5:**
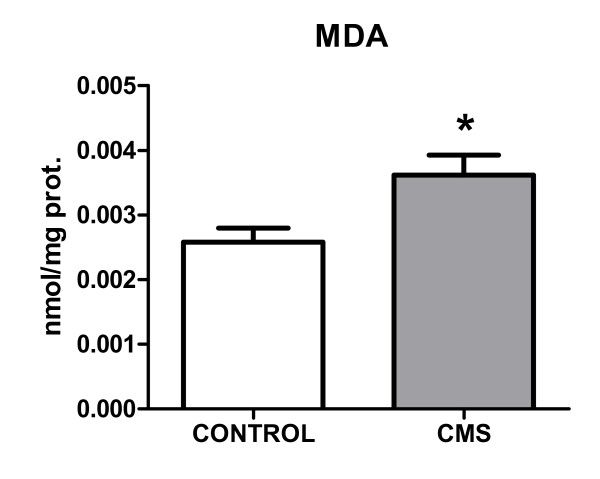
**Lipid peroxidation in brain after CMS: levels of malondialdehyde (MDA; a marker of reactive oxygen species attack and resultant lipid peroxidation) in control rats and after CMS exposure in brain cortex**. Data are mean ± SEM of 8-10 rats per group. * p < 0.05 (two-tailed t-test).

Although neither inducible nitric oxide synthase (iNOS) expression nor stable metabolites of nitric oxide (nitrites) levels were modified in brain cortex after 21 days of CMS (data not shown), we decided to study possible (COX-2- and cytokine-induced) oxidative/nitrosative damage after stress. As a final index of this type of damage that could be affected by CMS, we measured the accumulation of the lipid peroxidation marker malondialdehyde (MDA) in brain prefrontal cortex of the different groups of rats. MDA increased after CMS exposure (Figure 5).

### 5.- Effects of intestinal decontamination on CMS-induced inflammatory and oxidative/nitrosative damage

In order to evaluate whether the source of LPS (and subsequent TLR-4 activation) were bacteria translocated from the digestive tract, the effects of intestinal decontamination was assessed in our experimental setting. Antibiotic (ATB) decontamination decreased both stress-induced LPS and LBP increases in plasma (Table 1).

**Table 1:** Antibiotic intestinal decontamination (ATB) effect on stress-induced inflammatory, anti-inflammatory and oxidative/nitrosative parameters in control and CMS-exposed rats.

	Control	CMS	Control+ATB	CMS+ATB
***Plasma determinations***				

LPS (EU/mL)	0.2856 ± 0.027	0.3546 ± 0.006**	0.248 ± 0.022	0.3008 ± 0.016^#^

LBP (ng/mL)	799.8 ± 39.75	955.6 ± 35.57*	840.0 ± 19.52	804.2 ± 32.97^#^

***Brain determinations***				

TLR-4 (mRNA)	96.84 ± 2.618	109.8 ± 3.285**	102.5 ± 2.703	101.0 ± 1.278

TLR-4 (OD) (protein)	99.26 ± 4.455	116.9 ± 3.093**	88.09 ± 4.142	97.01 ± 3.162^##^

MD-2 (mRNA)	98.01 ± 2.575	108.4 ± 2.178**	91.74 ± 2.432	96.86 ± 3.912^#^

MD-2 (OD) (protein)	94.94 ± 2.977	108.6 ± 2.578*	102.6 ± 2.842	104.9 ± 4.381

NF-κB p65 Activity (% Control)	100.0 ± 4.571	85.48 ± 3.277*	96.73 ± 15,33	71.66 ± 3.1**

NF-κB p65 (mRNA)	101.8 ± 2.546	94.21 ± 2.193*	90.28 ± 2.052	88.16 ± 2.879

NF-κB p65 (OD) (protein)	100.6 ± 3.363	87.23 ± 3.554*	103.2 ± 4.530	99.43 ± 3.442^#^

IκBα (mRNA)	100.0 ± 4.286	118.7 ± 6.436*	95.55 ± 3.265	99.42 ± 5.101^#^

COX-2 (mRNA)	99.89 ± 5.056	137.2 ± 8.159**	124.6 ± 7.084	107.1 ± 6.181^#^

PGE_2_ (pg/mg prot.)	45.14 ± 6.485	78.69 ± 12.24*	48.58 ± 8.973	36.75 ± 7.877^#^

15d-PGJ_2_ (pg/mg prot.)	83.45 ± 13.99	42.00 ± 6.775*	83.28 ± 13.78	107.8 ± 21.68^#^

IL-1β (mRNA)	94.59 ± 4.000	114.0 ± 2.318**	95.91 ± 9.424	91.35 ± 3.886^##^

MDA (nmol/mg prot.)	0.00279 ± 0.000256	0.00372 ± 0.000285*	0.00187 ± 0.000142	0.00242 ± 0.000344^##^

Data are means ± SEM of 8-10 rats per group; * p < 0.05; ** p < 0.01 *vs*. Control; ^# ^p < 0.05; ^## ^p < 0.01 *vs*. CMS. One-way ANOVA followed by the Newman-Keuls *post hoc *test.

The effects of decontamination on stressed animals extended to stress-induced TLR-4 and MD-2 up-regulation at protein and mRNA levels, and to all of the other inflammatory and oxidative parameters previously determined in brain tissue (Table 1). Interestingly, ATB decontamination prevented the CMS-induced decrease in anti-inflammatory 15d-PGJ_2 _levels in the brain (Table 1).

### 6.- Effects of CMS and intestinal decontamination on plasma corticosterone levels

**Figure 6:**
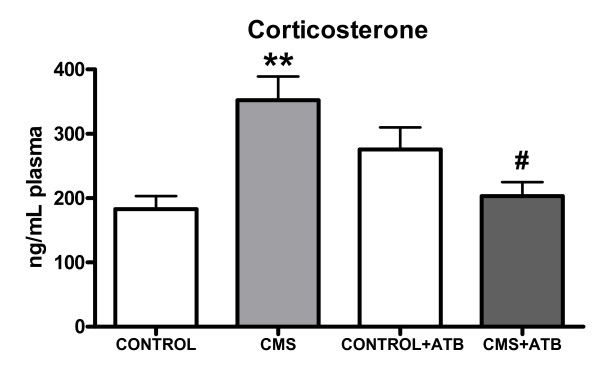
**Plasma corticosterone levels of control (non-stressed), CMS-exposed, control+intestinal antibiotic-decontamination (CONTROL+ATB) and CMS+ATB animals**. Data are mean ± SEM of 8-10 rats per group. ** p < 0.01 vs. Control group; #p < 0.05 vs. CMS group. One-way analysis of variance (ANOVA) followed by the Newman-Keuls *post hoc *test.

Chronic mild stress exposure increased plasma corticosterone levels when compared to the control group and to the group of rats subjected to CMS plus intestinal decontamination (CMS+ATB group). Antibiotic (ATB) treatment decreased corticosterone levels of chronically stressed rats (CMS+ATB group) and these CMS+ATB animals did not show differences in plasma corticosterone levels when compared to the control (non stressed) group, showing that intestinal decontamination inhibits the increase of corticosterone induced by the CMS protocol (Figure 6).

### 7.- Effects of CMS and intestinal decontamination on depressive-like behavior

**Figure 7:**
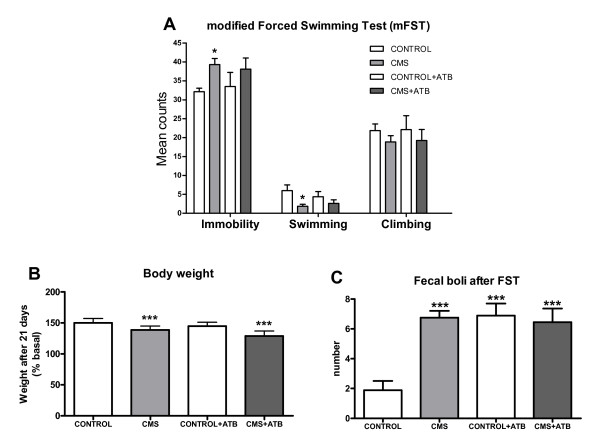
**Behavioral parameters (time in immobility, swimming and climbing, in seconds) during the modified forced swimming test (mFST) (A), weight change after 21 days of CMS exposure (B) and fecal boli (number) (C)**. Data are means ± SEM of 9-10 rats per group; * p < 0.05; ** p < 0.01 *vs*. Control. Two-way analysis of variance (ANOVA) followed by Bonferroni *post hoc *test.

After 21 days of the CMS protocol, separate groups of animals (n = 10) were exposed to the modified forced swimming test (mFST). Data show that after CMS exposure rats elicit a pro-depressive behavior (Figure 7A): immobility time is significantly increased in CMS, as shown by significant decreases in swimming time compared to the control group. Analysis of time climbing did not reveal significant differences between groups. Furthermore, weight loss and number of fecal boli were increased in CMS (Figure 7B&7C). However, in spite of the anti-inflammatory effects demonstrated in the brain by the antibiotic intestinal decontamination protocol used, ATB did not modify immobility or swimming behaviors after mFST in stressed animals (Figure 7A).

## Discussion

The present work points to a role for bacterial translocation and subsequent TLR-4 pathway stimulation in the neuroinflammation induced by an experimental model of depression. To our knowledge, our results demonstrate for the first time that the TLR-4 signaling pathway becomes activated in brain cortex of rats exposed to an animal model of depression. This activation occurs with increased levels of the pro-inflammatory cytokine IL-1β and of one of the main enzymatic sources of inflammatory and oxidative mediators, COX-2 and its product PGE_2_. Interestingly, after 21 days of CMS, the COX-derived anti-inflammatory mediator 15d-PGJ_2 _appears decreased. As a consequence of this misbalance and the resulting enhancement of inflammation and oxidation in brain cortex after CMS exposure, an increment in lipid peroxidation takes place.

In the search for a mechanistic explanation for the observed TLR-4 activation, experiments using antibiotic intestinal decontamination suggest a pivotal role for anaerobic Gram-negative bacteria translocation on TLR-4-signaling pathway activation after stress exposure in brain cortex of rats.

In accordance with other studies carried out in different models of stress exposure, including CMS, our data show that there is inflammatory and oxidative/nitrosative damage in the brain after CMS [5,38-40]. The increase of IL-1β mRNA levels detected in brain cortex also correlates with results obtained in previous studies [41-43]. This can be considered particularly significant, bearing in mind that this cytokine plays a central role in the sickness behavior detected in animals after LPS injection (LPS induces its release) and has been proposed as a possible actor involved in the pathophysiology of depression [6,44]. Moreover, the actions of IL-1β in the CNS include increases in the production of other pro-inflammatory cytokines which can stimulate enzymatic sources of oxidative and nitrosative mediators [45].

Apart from cytokines, other mediators such as bacterial endotoxin (i.e. LPS, which we are showing here also increased after CMS) rapidly induce COX-2 and PGE_2 _production [46,47]. The induction of COX-2 in the CNS by stress and the increase in the PGE_2 _levels in the brain cortex are well documented phenomena [48,49] of significant importance in experimental models of depression and in depressive disorders [50], bearing in mind that PGE_2_, in turn, stimulates production of pro-inflammatory cytokines, expression of COX-2 and, as a co-factor, activity of indoleamine 2,3-dioxygenase (IDO), which reduces levels of 5-HT, a hallmark of depression.

On the other hand, it has been previously shown that, during the production of prostaglandins, reactive oxygen species (ROS) are generated, which are a main cause of oxidative/nitrosative damage as has been shown to occur after CMS, leading to an increase in lipid peroxidation markers (increase in the amount of MDA) [51]. Although previous studies have revealed an increase in inducible nitric oxide synthase (iNOS) levels in the brain after acute and subacute stress protocols [5], after chronic exposure to a series of stressors of mild intensity (as occurs in CMS) the main isoform implicated is the constitutive, neuronal NOS (nNOS) isoform [52]. Thus, the increase in lipid peroxidation observed in the specific experimental setting used in the present study should be attributed mainly to cyclooxygenase-derived products.

Activation of the transcription factor nuclear factor *kappa *B (NF-κB) controls the transcription of many acute-phase proteins and inflammatory genes both in humans and rodents, and is one of the earliest events in the stress-inflammation response in the brain [53,54]. This transcription factor resides silent in the cytoplasm bound by an inhibitory protein, I *kappa *B *alpha *(IκBα). When a specific cellular pathway is stimulated, it produces phosphorylation and subsequent degradation of IκBα, activating NF-κB which translocates to cell nucleus where it recognizes specific DNA sequences in the promoter of target genes, among which are those that code for proteins involved in inflammation. Interestingly, no clear stimulation of NF-κB occurs in the brain cortex after CMS when its p65 subunit is analyzed. However, our results show that IκBα mRNA levels are increased after CMS. As it has been described to occur in other experimental settings, the increase in IκBα mRNA is an autoregulatory pathway switched on by NF-κB after prolonged stimulation as may be the case in CMS, thus restricting NF-κB action when chronically stimulated [55,56].

Having described some components of the inflammatory response in the brain cortex to CMS exposure, we focused on a search for possible external stressors stimulating this response, as recently reviewed by Kubera et al. [39]. All of the inflammatory parameters described up to this point can be induced by the Toll-like receptors (TLRs) pathway stimulation. TLRs, being the first line of defense against invading microorganisms, constitute the main agents of the innate immune response. Stimulation of TLRs causes an immediate defensive response, including the production of an array of antimicrobial peptides and inflammatory/oxidative mediators [37]. During the last several years numerous studies have appeared regarding the role of TLRs in the pathophysiology of diverse CNS diseases such as multiple sclerosis, Alzheimer's disease and brain ischemia [16,57,58]. Now, our results show for the first time increases in expression of and mRNA levels for Toll-like receptor 4 (TLR-4) in the brain cortex in an experimental model of depression in rodents. Additionally, we have also found that CMS induces protein expression and synthesis of MD-2, which is the molecule that confers lipopolysaccharide responsiveness to TLR-4 [59].

Taken as a whole, the results presented here suggest that TLR-4 could be an important regulatory factor in the consequences of chronic stress in the brain, and also support a possibility for pharmacological or genetic manipulations of this pathway - although to date the selective inhibition of TLR-4 has proved to be a difficult challenge [60] - in order to minimize oxidative and inflammatory damage in the CNS after stress and in stress-related psycho- and neuro-pathologies such as depression.

There are several studies exploring endogenous ligands that activate TLR-4 after brain damage (e.g. protein S100 or nuclear protein high-mobility group box 1 after cerebral ischemia, pro-inflammatory cytokines after brain trauma) [60]. However, knowledge about mechanisms that regulate TLR-4 activation in the brain in models of neuropsychiatric pathologies comes from previous studies based on stress exposure, which have shown increased intestinal permeability and a resultant bacterial translocation to the systemic circulation after stress exposure [21,22]. As a result, there are circulating Gram-negative enterobacteria, which are a major source of LPS and can activate brain TLR-4 inducing a neuroinflammatory response. In order to clarify the origin of stress-induced activation of the TLR-4 pathway in CMS, we studied LPS and its binding protein (LBP; which serves as a lipid transfer protein that facilitates the transportation of LPS to the recognition protein CD14 and to TLR-4) levels in plasma. Our results show that CMS exposure produces increases in both LPS and LBP plasma levels. Thus, it is possible that CMS is causing an intestinal dysfunction followed by bacterial translocation, as occurs in different stress models in rodents [22], with LPS (from those Gram-negative bacteria) being the reason for the TLR-4 activation. This proposed mechanism, known as "*leaky gut*", also takes place in depressed patients, and has been related to the inflammatory pathophysiology of major depressive disorder [23].

To assess, in our experimental setting, whether the source of LPS and the consequent TLR-4 pathway stimulation, are bacteria translocated from the gut, we examined the effects of intestinal decontamination on the stress-induced inflammatory and oxidative/nitrosative changes revealed above. We used a standard stringent protocol (streptomycin and penicillin G) for only intestinal decontamination. This protocol has been used because it has demonstrated to lack any neuroprotective or anti-inflammatory effects on the CNS when used in other related protocols [24,61]. By using this protocol, we can separate possible effects on the brain of the antibiotic used (i.e. the anti-neuroinflammatory effect of minocycline) from the effects caused by intestinal decontamination.

Our data show that animals subjected to CMS plus intestinal decontamination present a return to basal levels (control group values) for pro-inflammatory and oxidative/nitrosative parameters previously analyzed, including LPS and LBP plasma concentrations and TLR-4 and MD-2 expression and mRNA levels.

In this vein, of special relevance is the finding that antibiotic intestinal decontamination promotes decreases in IL-1β and COX-2/PGE_2 _in brain cortex. This result supports the notion that LPS from translocated bacteria stimulates TLR-4, and in that way produces the increases in IL-1β and COX-2/PGE_2 _levels in the CNS previously detected. More interestingly, intestinal decontamination is able to restore the disbalance between COX-derived inflammatory (PGE_2_) and anti-inflammatory (15d-PGJ_2_) components in the brain.

Our results also indicate that plasma corticosterone levels are increased after 21 days of CMS when compared with the control group, showing that even after this chronic stress exposure the hypothalamic-pituitary-adrenal (HPA) axis of these animals remains functioning. Additionally, it has been previously demonstrated that LPS stimulates the HPA axis [62] and thus, it is conceivable that the increase in the corticosterone levels after CMS could be caused, at least in part, by the increase in LPS levels detected here and not only by the stressors themselves. Supporting this idea, the intestinal decontamination that decreases LPS after CMS, also decreases plasma corticosterone levels, again supporting the role of intestinal bacteria as a source for the LPS detected in our study.

The effects of intestinal decontamination on depressive-like behavior were analyzed using a modified forced swimming test based on the method described by Porsolt [32], measuring behavioral despair. In spite of its anti-inflammatory effects after decreasing LPS levels, antibiotic decontamination failed to reverse the depressive-like behavior induced by CMS, which indicates a role for LPS-induced neuroinflammation after CMS without (at this level) behavioral consequences. Nonetheless, the fact that CMS-induced neuroinflammation is reversed by antibiotic intestinal decontamination is particularly relevant because neuroinflammation is considered an important biological event that might increase the risk of major depressive episodes much like more traditional psychosocial factors [6]. Further studies using mixed protocols of experimental depression plus infective or inflammatory agents would aid in explaining the role of comorbid depression in inflammatory or immune-related pathologies.

The results presented here are in line with a hypothesis recently presented [38] according to which, external stressors to the brain, such as LPS, may up-regulate immune receptors such as TLR-4 that, in turn, may aggravate neuroinflammation due to locally produced internal stressors (prostanoids, some cytokines, transcription factors) thus causing a superinduction of (neuro)inflammatory responses.

## Conclusions

**Figure 8:**
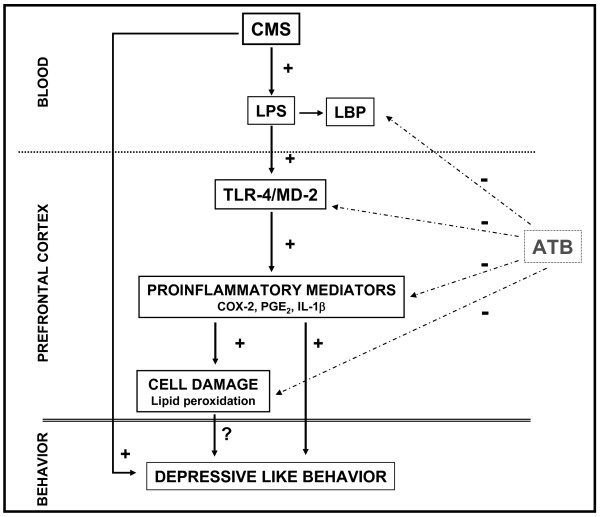
**Schematic representation of the results obtained from and the effects of antibiotic intestinal decontamination (ATB: intestinal antibiotic decontamination)**. See text for abbreviations.

In conclusion, our results suggest that LPS from bacterial translocation is responsible, at least in part, for the TLR-4 activation found in the brain after chronic mild stress exposure which leads to the release of inflammatory mediators in the CNS (including IL-1β and COX-2) (Figure 8). In addition, antibiotic intestinal decontamination decreases LPS systemic levels and neuroinflammation showing a possible protective role of antibiotic decontamination in stress-related conditions and offering a potential therapeutic target for the adjuvant treatment of depression.

## Competing interests

The authors declare that they have no competing interests.

## Authors' contributions

IG contributed to acquisition, analysis and interpretation of data; BGB contributed to acquisition, analysis and interpretation of data, drafting the manuscript and revising it critically; JLMM contributed to analysis and interpretation of data and revising the manuscript critically; LB and EB contributed to acquisition, analysis and interpretation of CMS model and behavioural data; JAM and JRC revised the manuscript critically; and JCL contributed to conception and design, drafting the manuscript and revising it critically for important intellectual content. All authors have given final approval of the version to be published.
